# Effect of Lemborexant‐Based Sleep Medication Formulary on Benzodiazepine Reduction and Clinical Outcomes: A Single‐Center Retrospective Study

**DOI:** 10.1002/npr2.70054

**Published:** 2025-09-08

**Authors:** Shunya Aoki, Katsutoshi Takada, Tatsuru Sugama, Mitsugi Kimiwada, Tatsuya Hoshino, Kaori Koike, Hirokazu Akada, Takahisa Saiga, Shigeki Sato, Ryosuke Shinkai, Yukihiro Shibata, Takashi Tomita

**Affiliations:** ^1^ Pharmaceutical Department Japanese Red Cross Narita Hospital Narita Chiba Japan; ^2^ Nursing Department Japanese Red Cross Narita Hospital Narita Chiba Japan; ^3^ Department of Psychiatry Japanese Red Cross Narita Hospital Narita Chiba Japan; ^4^ Department of Pharmaceutical Sciences School of Pharmacy at Narita International University of Health and Welfare Narita Chiba Japan; ^5^ Department of Pharmacy International University of Health and Welfare Mita Hospital Minato‐ku Tokyo Japan

**Keywords:** accidental falls, delirium, formulary, hypnotics and sedatives, lemborexant

## Abstract

Benzodiazepine and non‐benzodiazepine hypnotics (Z‐drugs) are known risk factors for adverse events, including delirium and falls. Although formularies are intended to promote appropriate prescribing, few comprehensive studies have assessed their clinical impact in the context of sleep medications. This study aimed to evaluate changes in hypnotic prescribing patterns and associated clinical outcomes following the implementation of a sleep medication formulary. A psychiatric liaison team developed and implemented a formulary in April 2024, recommending lemborexant as the first‐line treatment and eszopiclone as the second‐line option. This single‐center, retrospective study compared patients admitted and discharged during the 12 months before (April 2023 to March 2024; *n* = 12 633) and after (April 2024 to March 2025; *n* = 12 931) implementation. Outcome measures included monthly prescription volumes, diazepam equivalents, use in clinical pathways and prescription sets, delirium incidence, nighttime falls, and length of hospital stay. Statistical analyses were performed using the Mann–Whitney *U*‐test and Fisher's exact test. Following implementation, prescription volumes of lemborexant and eszopiclone increased significantly, whereas diazepam equivalents decreased from 10 682 mg to 4117 mg. All 104 clinical pathways and prescription sets previously using benzodiazepine hypnotics or Z‐drugs were converted to lemborexant. Monthly delirium cases declined from 12.5 to 8.0, and the proportion of nighttime falls among patients receiving benzodiazepine hypnotics or Z‐drugs decreased from 24.0% to 11.5%. The median hospital stay also decreased from 8 to 7 days. These findings suggest that formulary implementation effectively optimized hypnotic prescribing and contributed to improved clinical outcomes and patient safety in an acute care setting.

## Abstract 2

Lemborexant‐based sleep medication formulary reduced benzodiazepine use by 61%, decreased monthly delirium cases and nighttime falls, while shortening hospital stays. This demonstrates that formulary implementation effectively optimizes hypnotic prescribing and improves patient safety outcomes.

## Graphical abstract

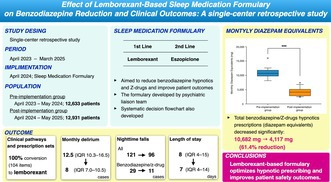

## 1 Introduction

A formulary is a list of medications and accompanying operational guidelines selected by healthcare institutions based on considerations of efficacy, safety, and cost‐effectiveness, thereby enabling appropriate prescribing by non‐specialist physicians [1, 2, 3, 4]. This system facilitates standardized therapeutic approaches while reducing healthcare costs and enhancing the overall quality of care [2, 3]. Formularies in Japan adhere to these principles [4], modeled after frameworks such as the American Society of Health‐System Pharmacists (ASHP) “Guidelines on the Pharmacy and Therapeutics Committee and the Formulary System” [2] and the National Institute for Health and Care Excellence (NICE) “Developing and updating local formularies” [3]. However, their implementation in Japan remains limited [4].

Benzodiazepine and non‐benzodiazepine hypnotics (Z‐drugs: zolpidem, zopiclone, eszopiclone) have been identified as risk factors for adverse events, including delirium and falls [5, 6, 7, 8, 9, 10], which may lead to prolonged hospitalization and increased healthcare expenditures [11, 12, 13, 14, 15, 16, 17]. Although recent healthcare reforms in Japan have targeted reductions in such prescriptions, real‐world clinical practice has shown limited progress in this regard [18, 19]. In contrast, orexin receptor antagonists have demonstrated lower rates of adverse events [20, 21], positioning them as favorable alternatives, especially relevant in Japan, where benzodiazepine prescribing remains notably high [18]. Although formularies may hold promise for facilitating such transitions [22], studies systematically evaluating the formulary implementation of sleep medications remain limited.

The present study aimed to assess the clinical impact of formulary implementation for sleep medications, with a particular focus on the reduction of benzodiazepine hypnotics and Z‐drugs and its association with delirium, falls, and length of hospital stay.

## 2 Methods

### 2.1 Study Design

This single‐center, retrospective observational study evaluated changes in prescription patterns and clinical outcomes before and after the implementation of a sleep medication formulary. The study period spanned from April 2023 to March 2025 and included patients admitted to and discharged from general wards during this timeframe. Patients admitted to psychiatric and pediatric wards were excluded. Psychiatric wards were excluded due to their focus on acute psychiatric disorders, where benzodiazepine hypnotics and Z‐drugs are often clinically indicated [23, 24, 25]. Pediatric wards were excluded because benzodiazepine hypnotics, Z‐drugs, and orexin receptor antagonists are off‐label for pediatric use. The period from April 2023 to March 2024 was defined as the “pre‐implementation group,” and the period from April 2024 to March 2025 was defined as the “post‐implementation group.” The effects of the formulary were evaluated by comparing these two groups. The pre‐implementation group comprised 12 633 patients, whereas the post‐implementation group included 12 931 patients.

### 2.2 Study Setting

The study was conducted at Narita Red Cross Hospital, a general acute care hospital under Japan's Diagnosis Procedure Combination (DPC) system.

### 2.3 Psychiatric Liaison Team

The hospital maintains a psychiatric liaison team to address psychiatric symptoms in patients with physical illnesses [26]. This multidisciplinary team includes physicians, nurses, pharmacists, clinical psychologists, and psychiatric social workers, who conduct weekly ward rounds and interdisciplinary conferences.

### 2.4 Sleep Medication Formulary

**FIGURE 1:**
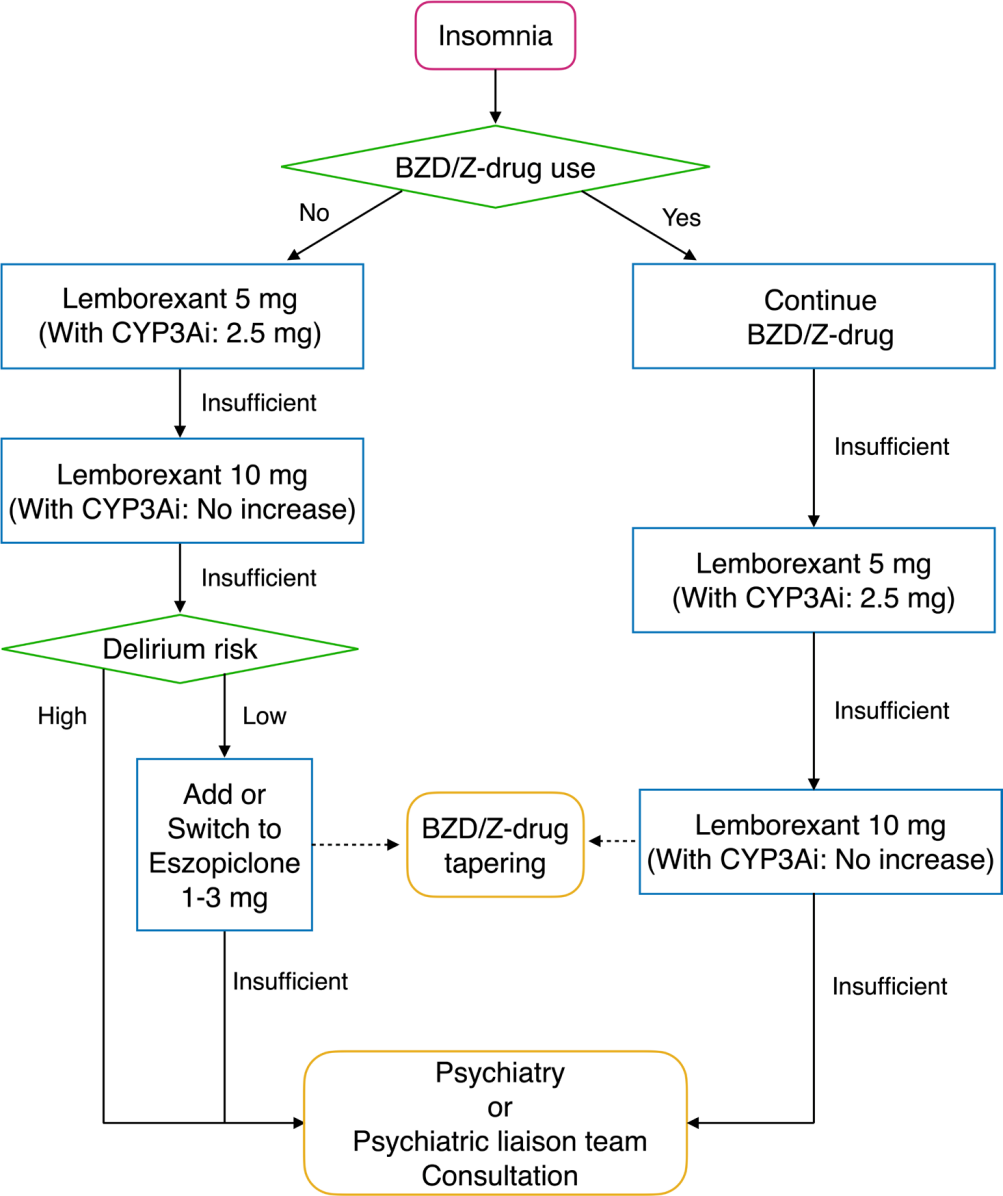
Decision algorithm for sleep medication prescribing following formulary implementation. The flowchart guides appropriate hypnotic selection based on current benzodiazepine or non‐benzodiazepine hypnotic (Z‐drug) use and delirium risk assessment. Dosing precautions and CYP3A inhibitor interactions follow Japanese package insert. Abbreviations: BZD, benzodiazepine; Z‐drug, non‐benzodiazepine hypnotic; CYP3Ai, cytochrome P450 3A inhibitor.

The sleep medication formulary was developed by the psychiatric liaison team based on published evidence [5, 7, 8, 20, 27, 28, 29, 30]. Implemented in April 2024, the formulary was disseminated hospital‐wide as part of mandatory staff training conducted by the Medical Safety Committee. Lemborexant, an orexin receptor antagonist, was selected as the first‐line agent, and eszopiclone, a Z‐drug, as the second‐line agent. A prescription flowchart (Figure 1) was created to guide non‐specialist physicians in medication selection. Dosing was based on the Japanese prescribing information: lemborexant 5 mg as the standard dose, reduced to 2.5 mg when co‐administered with cytochrome P450 3A inhibitors (CYP3Ai) [31]. For cases in which the recommended agents were inadequate or patients were deemed high‐risk for delirium—evaluated using the Delirium Team Approach (DELTA) program [32]—consultation with the psychiatric liaison team or psychiatry department was advised.

### 2.5 Hospital‐Adopted Hypnotics

The hospital's adopted hypnotics were categorized into three groups: benzodiazepines, Z‐drugs, and orexin receptor antagonists (Table 2). Although classified as an anxiolytic (N05BA) by the World Health Organization (WHO) [33], etizolam was included as a benzodiazepine hypnotic due to its routine use as a sleep aid in Japan [34].

### 2.6 Outcome Measures

To assess the effects of formulary implementation, the following outcome measures were retrospectively analyzed for both the pre‐ and post‐implementation groups. Data were extracted from the electronic medical record system “HAPPY ACTIS” (Canon Medical Systems Corporation, Tochigi, Japan) for patient characteristics, prescription data, delirium cases, and length of hospital stay. Nighttime fall data were retrieved from the hospital incident reporting system “Safe Master” (SafeMaster Corporation, Fukuoka, Japan).

#### 2.6.1 Monthly Prescription Volumes

Monthly prescription volumes for each hypnotic were compiled and compared between groups.

#### 2.6.2 Monthly Diazepam Equivalents

Monthly prescription volumes for benzodiazepine hypnotics and Z‐drugs were converted to 5 mg equivalents of diazepam, based on Inada et al. [35], allowing standardized comparisons across medications.

#### 2.6.3 Clinical Pathways and Prescription Sets

The types of hypnotics used in clinical pathways and predefined prescription sets were analyzed for both groups.

#### 2.6.4 Monthly Delirium Cases

Monthly delirium cases were compiled and compared between the groups. Delirium cases were defined as cases diagnosed by specialists based on internal referral letters to psychiatry. Monthly consultations with the psychiatric liaison team were also recorded.

#### 2.6.5 Nighttime Falls

Incident reports occurring between 22:00 and 06:59 h were compiled for both groups. This timeframe was selected based on prior reports indicating increased hypnotic effects and the strongest fall risk during these hours [36]. Cases were stratified by hypnotic use and type.

#### 2.6.6 Length of Hospital Stay

Length of hospital stay was compared between groups.

### 2.7 Statistical Analysis

**FIGURE 2:**
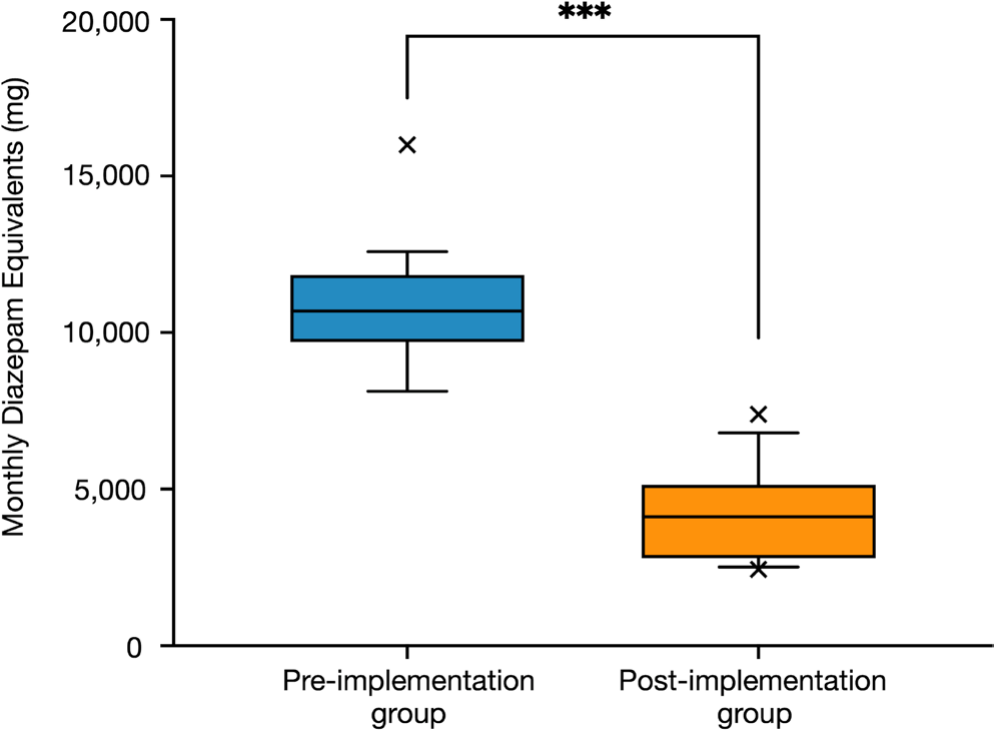
Monthly diazepam equivalents of benzodiazepine and non‐benzodiazepine hypnotics (Z‐drugs) decreased significantly following a sleep medication formulary implementation. Pre‐implementation group: April 2023 to March 2024 (*n* = 12 months); Post‐implementation group: April 2024 to March 2025 (*n* = 12 months). Data presented as median with interquartile range (IQR). Whiskers represent 1.5× IQR, and outliers are plotted individually as crosses (×). ***p < 0.001 by Mann–Whitney *U*‐test.

**FIGURE 3:**
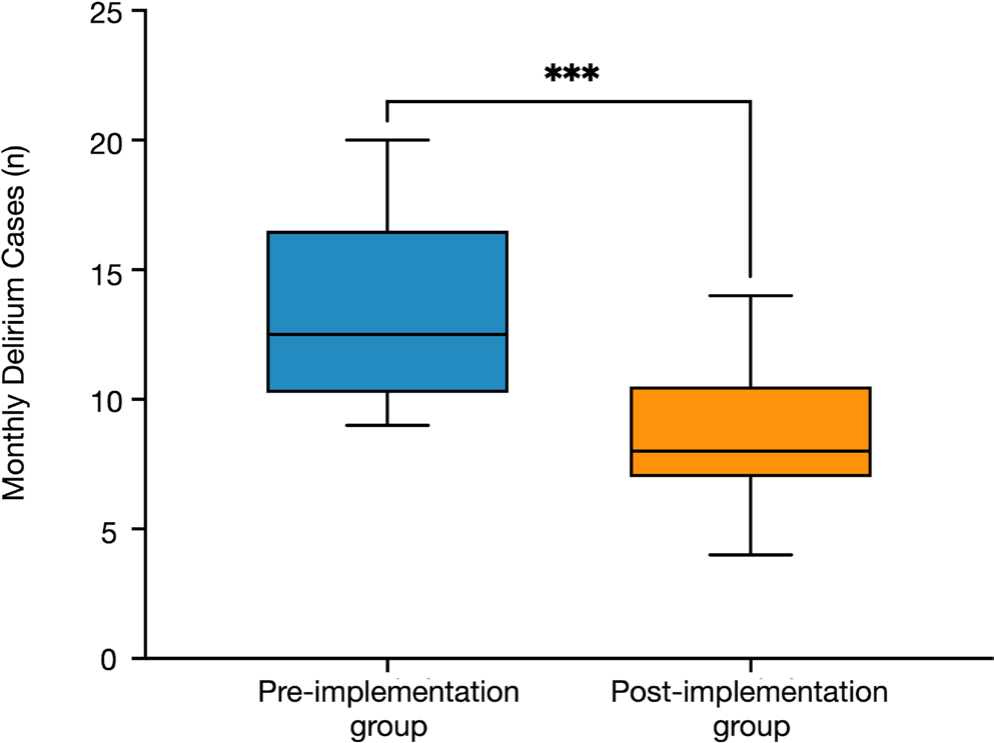
Monthly delirium cases decreased significantly following a sleep medication formulary implementation. Pre‐implementation group: April 2023 to March 2024 (*n* = 12 months); Post‐implementation group: April 2024 to March 2025 (*n* = 12 months). Data presented as median with interquartile range (IQR). Whiskers represent 1.5× IQR. ***p < 0.001 by Mann–Whitney *U*‐test.

Statistical comparisons were conducted as follows: The Mann–Whitney *U*‐test was used for monthly prescription volumes, diazepam equivalents, delirium cases, and length of hospital stay. Fisher's exact test was used to compare nighttime falls. A two‐tailed *p*‐value < 0.05 was considered statistically significant. Figures 2 and 3 present data as median with interquartile range (IQR), with whiskers representing 1.5× IQR and outliers plotted individually. All statistical analysis was performed using GraphPad Prism 10 for Mac (Version 10.4.2 (534), GraphPad Software Inc., San Diego, CA, USA).

### 2.8 Ethical Considerations

This study was approved by the Narita Red Cross Hospital Ethics Committee (Approval number: 962‐01) and complied with the Declaration of Helsinki. An opt‐out method was employed, and study details were publicly disclosed on the hospital's website. Patient data were anonymized and handled in accordance with relevant personal data protection laws and institutional ethical guidelines.

## 3 Results

### 3.1 Patient Characteristics

Patient characteristics are presented in Table 1 (*n* = 12 633 for the pre‐implementation group; *n* = 12 931 for the post‐implementation group).

**TABLE 1:** Patient characteristics and hypnotic prescriptions.

Variables	Pre‐implementation group (*n* = 12 633)	Post‐implementation group (*n* = 12 931)
Age (year), mean (SD)	64.8 (19.2)	65.5 (18.9)
Age (year), *n* (%)		
< 19	316 (2.5)	326 (2.5)
20–39	1382 (10.9)	1274 (9.9)
40–59	2147 (17.0)	2201 (17.0)
60–79	5999 (47.5)	6180 (47.8)
≥ 80	2789 (22.1)	2950 (22.8)
Male, *n* (%)	7122 (56)	7337 (57)
Clinical department, *n* (%)		
Internal medicine	5303 (42.0)	5804 (44.9)
General surgery	1219 (9.6)	1245 (9.6)
Orthopedic surgery	1184 (9.4)	1177 (9.1)
Obstetrics and gynecology	1102 (8.7)	1012 (7.8)
Urology	1048 (8.3)	1036 (8.0)
Otorhinolaryngology	614 (4.9)	638 (4.9)
Neurosurgery	592 (4.7)	572 (4.4)
Neurology	496 (3.9)	492 (3.8)
Plastic surgery	262 (2.1)	218 (1.7)
Thoracic surgery	230 (1.8)	181 (1.4)
Ophthalmology	198 (1.6)	159 (1.2)
Cardiovascular surgery	179 (1.4)	185 (1.4)
Oral surgery	164 (1.3)	175 (1.4)
Others	42 (0.3)	36 (0.3)
Hypnotic prescriptions, *n* (% of total hypnotic users)	3768	3548
Benzodiazepines	867 (23)	381 (10.7)
Etizolam	514 (13.6)	206 (5.8)
Brotizolam	173 (4.6)	92 (2.6)
Nitrazepam	59 (1.6)	23 (0.6)
Flunitrazepam	53 (1.4)	34 (1.0)
Rilmazafone	38 (1.0)	17 (0.5)
Triazolam	30 (0.8)	9 (0.3)
Non‐benzodiazepines (Z‐drugs)	605 (16.1)	253 (7.1)
Zolpidem	520 (13.8)	170 (4.8)
Zopiclone	47 (1.2)	1 (0.03)
Eszopiclone	38 (1.0)	82 (2.3)
Orexin receptor antagonists	2296 (60.9)	2914 (82.1)
Lemborexant	2127 (56.4)	2804 (79)
Suvorexant	169 (4.5)	110 (3.1)

### 3.2 Monthly Prescription Volumes

Monthly prescription volumes of the recommended medications, lemborexant and eszopiclone, increased significantly (*p* = 0.0009 and *p* < 0.0001, respectively). In contrast, the prescription volumes of most benzodiazepine hypnotics (etizolam, brotizolam, nitrazepam 1% granule, flunitrazepam 1 mg) and Z‐drugs (zolpidem 5 mg, zopiclone) decreased significantly (Table 2). The use of suvorexant, a non‐recommended orexin receptor antagonist, also decreased significantly.

**TABLE 2:** Monthly prescription volumes.

Hypnotics	Pre‐implementation group mean (SD)	Post‐implementation group mean (SD)	*p*
Benzodiazepines			
Etizolam 0.5 mg	2541.3 (372.1)	483.5 (368.4)	**< 0.0001**
Etizolam 1 mg	438.6 (74.6)	106.3 (102.6)	**< 0.0001**
Brotizolam 0.25 mg	357.0 (55.2)	221.2 (65.7)	**< 0.0001**
Nitrazepam 5 mg	69.4 (33.8)	52.3 (45.5)	0.1925
Nitrazepam 1% granule	7.0 (23.2)	—	—
Flunitrazepam 1 mg	53.6 (42.6)	15.5 (16.8)	**0.0398**
Flunitrazepam 2 mg	43.6 (36.3)	27.8 (22.1)	0.3685
Triazolam 0.25 mg	37.0 (33.9)	36.8 (21.2)	0.5604
Rilmazafone 1 mg	17.7 (15.6)	12.5 (18.8)	0.2101
Rilmazafone 2 mg	11.9 (20.2)	7.2 (9.2)	0.7478
Non‐benzodiazepines (Z‐drugs)			
Zolpidem 5 mg	389.6 (90.4)	166.8 (48.6)	**< 0.0001**
Zolpidem 10 mg	74.0 (34.9)	55.8 (45.2)	0.1827
Zopiclone 7.5 mg	46.1 (34.4)	—	—
Zopiclone 10 mg	30.0 (17.9)	1.6 (3.2)	**< 0.0001**
Eszopiclone 2 mg	5.7 (13.6)	102.7 (53.0)	**< 0.0001**
Orexin receptor antagonists			
Lemborexant 5 mg	2319.9 (423.3)	3019.0 (388.5)	**0.0009**
Suvorexant 15 mg	175.3 (46.4)	101.9 (50.6)	**0.0022**
Suvorexant 20 mg	96.4 (55.3)	25.0 (28.2)	**0.0009**

*Note:* Bold numbers indicate *p* < 0.05 by Mann–Whitney *U*‐test.

### 3.3 Monthly Diazepam Equivalents

Monthly diazepam equivalent doses for all benzodiazepine hypnotics and Z‐drugs decreased significantly, from 10 682 mg to 4117 mg (*p* < 0.0001) (Figure 2).

### 3.4 Clinical Pathways and Prescription Sets

In the pre‐implementation group, all 104 clinical pathways and prescription sets that included hypnotics used benzodiazepine hypnotics or Z‐drugs. In the post‐implementation group, all 104 items were fully replaced with lemborexant 5 mg (Table S1).

### 3.5 Monthly Delirium Cases

The median number of monthly delirium cases decreased significantly from 12.5 to 8.0 (*p* = 0.0005) (Figure 3). Psychiatric liaison team consultations related to delirium showed a decreasing trend, although the difference was not statistically significant (4.5 to 2 consultations; *p* = 0.0519) (Figure S1).

### 3.6 Nighttime Falls

The number of nighttime falls (22:00–06:59) decreased from 121 to 96. Among these, the number of falls in patients who had taken benzodiazepine hypnotics or Z‐drugs on the day of the incident decreased significantly (29/121 vs. 11/96, *p* = 0.0218). In contrast, falls among patients who had taken orexin receptor antagonists showed no significant differences (13/121 vs. 16/96, *p* = 0.2312) (Table 3).

**TABLE 3:** Nighttime falls (22:00–06:59) by hypnotic medication type.

Hypnotics	Pre‐implementation group	Post‐implementation group	*p*
	(*n* = 121)	(*n* = 96)	
Benzodiazepine/Non‐benzodiazepine hypnotics (Z‐drugs) users	29	11	**0.0218**
Orexin receptor antagonists users	13	16	0.2312

*Note:* Bold numbers indicate *p* < 0.05.

### 3.7 Length of Hospital Stay

The median length of hospital stay was significantly decreased from 8 to 7 days (*p* < 0.0001) (Table 4).

**TABLE 4:** Length of hospital stay.

Length of hospital stay	Pre‐implementation group	Post‐implementation group	*p*
	*n* = 12 633	*n* = 12 931	
Median, days (IQR)	8 (4–15)	7 (4–14)	**< 0.0001**
Mean, days (SD)	12.8 (16.4)	11.9 (14.9)	
Distribution, *n* (%)			
0–9	7579 (60.0)	8088 (62.5)	
10–19	2836 (22.4)	2759 (21.3)	
20–29	961 (7.6)	986 (7.6)	
30–39	527 (4.2)	451 (3.5)	
40–49	278 (2.2)	262 (2.0)	
50–59	163 (1.3)	139 (1.1)	
60–69	91 (0.7)	86 (0.7)	
70–79	65 (0.5)	71 (0.5)	
80–89	44 (0.3)	28 (0.2)	
90–99	22 (0.2)	16 (0.1)	
≥ 100	67 (0.5)	45 (0.3)	

*Note:* Bold numbers indicate *p* < 0.05 by Mann–Whitney *U*‐test.

## 4 Discussion

This study evaluated the clinical impact of a lemborexant‐based sleep medication formulary developed by a psychiatric liaison team. The findings demonstrated successful switching to recommended medications, significant reductions in benzodiazepine hypnotic and Z‐drug prescriptions, and substantial improvements in clinical outcomes, including reductions in delirium incidence, nighttime falls, and length of hospital stay. These findings suggest that implementing a sleep medication formulary contributes to optimizing prescribing practices and enhancing medical safety.

The formulary implementation promoted prescribing shifts toward recommended agents—lemborexant and eszopiclone—while substantially reducing the use of benzodiazepine hypnotics and Z‐drugs. A 61.4% decrease in monthly diazepam equivalents highlighted the extent of reduced high‐risk medication use. Although formulary systems have been introduced in Japan for other medication classes [37, 38], their application to hypnotics has been limited [39]. Given the known risks of benzodiazepine hypnotics and Z‐drugs—such as dependence, falls, and delirium [5, 6, 7, 8, 9, 10, 17, 40]—prescription reduction remains essential. Despite repeated healthcare reforms since 2012 [19], Japan continues to report higher prescription rates of these agents compared with other developed countries [18].

Based on evidence suggesting that formulary use influences prescribing behavior [22, 37, 38, 41], a sleep medication formulary was developed using rigorous, evidence‐based evaluation. Lemborexant, the first‐line agent, has been recommended in network meta‐analyses for its efficacy and tolerability [20, 30]. Compared with conventional benzodiazepines, orexin receptor antagonists offer lower risks of dependence, falls, and delirium [20, 30]. Some studies have even suggested that orexin receptor antagonists may improve delirium [21, 42, 43]. Eszopiclone, designated as the second‐line agent, was also supported by meta‐analyses for efficacy and tolerability [20, 30], and was found to pose a relatively lower fall risk among Z‐drugs. However, eszopiclone still carries higher risks compared with orexin receptor antagonists [20, 30], justifying its secondary placement.

The sleep medication formulary was disseminated hospital‐wide through mandatory e‐learning, and this combined approach—formulary development and staff education—led to institution‐wide behavioral change in hypnotic prescribing within a short period. Clinical pathways and prescription sets were also updated, resulting in complete replacement of benzodiazepine hypnotics and Z‐drugs with lemborexant. Clinical pathways are important for standardized, efficient medical care delivery [44]; their revisions typically require considerable time due to individual physician discretion. However, this coordinated implementation enabled rapid change. As a result, most prescriptions of high‐risk hypnotics were significantly reduced, with the change reflected in a substantial decrease in monthly diazepam equivalents.

Formulary implementation also demonstrated multiple clinical benefits. Delirium cases decreased significantly, and although psychiatric liaison consultations showed only a non‐significant decreasing trend, the overall reduction suggests improved patient safety. Delirium is associated with poor prognosis, higher mortality, longer hospital stays, and increased healthcare costs [13, 14, 15, 16]. Benzodiazepine hypnotics and Z‐drugs are known contributors to delirium [6], underscoring the clinical significance of prescription reduction.

In terms of fall risk, nighttime falls among patients using benzodiazepine hypnotics or Z‐drugs declined significantly from 24.0% to 11.5%, emphasizing the formulary's contribution to safer prescribing. Although a numerical increase in falls was observed among orexin receptor antagonist users (10.7% to 16.7%), this difference was not statistically significant and likely reflects the increased overall use rather than increased individual risk. Because falls can prolong hospitalization and increase healthcare costs, regardless of severity [11, 12], reducing high‐risk hypnotic use directly supports improved patient outcomes [6, 10].

The significant reduction in the length of hospital stay further supports the clinical and economic benefits of formulary implementation. Reduced delirium and fall events likely contributed to shorter hospitalizations. In Japan's DPC system—based on models such as diagnosis‐related groups (DRG)/prospective payment systems (PPS) in the United States and Germany [45, 46]—a shorter hospital stay translates directly into increased hospital revenue. Therefore, the observed reductions have both safety and financial implications.

This study has several limitations. First, as a single‐center, retrospective study, its findings may not be generalizable without multicenter validation. Second, delirium cases were identified through referral documentation, and fall data relied on incident reports, which may have led to underreporting of hypoactive delirium or minor fall events. Future prospective studies should use validated delirium screening tools such as the Confusion Assessment Method (CAM) [47] and objective fall detection methods. Third, lemborexant is not available in generic form and remains costly. Future studies should include economic evaluations using measures such as quality‐adjusted life‐years (QALYs) and incremental cost‐effectiveness ratio (ICER) [4]. Rather than focusing solely on pharmaceutical costs, broader evaluations of total medical costs are necessary to assess economic impact [1]. Fourth, delirium, falls, and length of hospital stay are influenced by multiple factors, including age, comorbidities, illness severity, other medications, and environmental conditions [5, 6, 10, 25, 48, 49], which were not controlled in this study. Future studies should apply multivariate analyses to account for these confounders.

Finally, formulary recommendations must be updated regularly in response to new evidence and emerging medications. Future studies should address newly approved hypnotics and continuously revise the formulary to ensure its relevance and effectiveness.

This study suggests that sleep medication formularies are an effective mechanism for converting physician‐dependent hypnotic prescribing into standardized, evidence‐based practice. In general wards, their utility is especially high, serving as institutional frameworks that ensure prescribing consistency and enhance medication safety. A recent study emphasized the role of psychiatric teams in improving outcomes for patients with dementia and physical illnesses [50]. Our findings extend this concept, suggesting that sleep medication formularies led by psychiatric liaison teams improve prescribing practices and contribute to enhanced safety, clinical outcomes, and hospital efficiency through evidence‐based management. Given the ongoing risks associated with benzodiazepine hypnotics and Z‐drugs, further reductions in their use are warranted, particularly at the community level. When patients prescribed these medications in outpatient settings are admitted to acute care hospitals, continuation is often necessary due to potential dependence and withdrawal symptoms. Therefore, coordinated community‐wide interventions—such as promoting local formulary operations—are essential, as demonstrated in prior successful implementations [37].

## 5 Conclusions

This study suggests that implementing a lemborexant‐based sleep medication formulary represents an effective institutional strategy for optimizing hypnotic prescribing and improving patient safety outcomes. The formulary led to significant reductions in benzodiazepine hypnotic and Z‐drug use and was associated with lower rates of delirium, nighttime falls, and length of hospital stay. These findings support the utility of psychiatric liaison team‐led formulary initiatives in enhancing both medication safety and healthcare efficiency within acute care settings.

## Author Contributions

Shunya Aoki conceived this study, led the sleep medication formulary development, performed data analysis, and wrote the manuscript. Shunya Aoki, Mitsugi Kimiwada, Tatsuya Hoshino, Kaori Koike, Hirokazu Akada, Shigeki Sato, and Takahisa Saiga developed and implemented the sleep medication formulary. Kaori Koike, Hirokazu Akada, Shigeki Sato, and Takahisa Saiga diagnosed delirium patients. Shunya Aoki, Tatsuya Hoshino, Kaori Koike, and Takahisa Saiga conducted patient consultations as members of the psychiatric liaison team. Tatsuru Sugama performed data extraction. Katsutoshi Takada, Ryosuke Shinkai, Yukihiro Shibata, and Takashi Tomita supervised this study. All authors read and approved the final manuscript.

## Ethics Statement

This study was approved by the Narita Red Cross Hospital Ethics Committee (Approval number: 962‐01) and complied with the Declaration of Helsinki. Patient data were anonymized and handled in accordance with relevant personal data protection laws and institutional ethical guidelines.

## Consent

An opt‐out method was employed, and study details were publicly disclosed on the hospital's website.

## Conflicts of Interest

The authors declare no conflicts of interest.

## Supporting information


**Figure S1:** Monthly delirium consultations to the psychiatric liaison team showed a decreasing trend following sleep medication formulary implementation.

**Table S1:** Changes in hypnotic selection in clinical pathways and prescription sets.

## Data Availability

This study used an opt‐out approach, and participants did not provide explicit informed consent for open data sharing. As such, the data are not publicly available to protect participant privacy. However, anonymized data may be made available upon reasonable request to the corresponding author via e‐mail for legitimate research purposes.
