# Supplementary material for: Effect of Lemborexant‐Based Sleep Medication Formulary on Benzodiazepine Reduction and Clinical Outcomes: A Single‐Center Retrospective Study
Source: Neuropsychopharmacol Rep. 2025 Sep 8;45(3):e70054. doi: 10.1002/npr2.70054 (PMC12416910; doi:10.1002/npr2.70054)
Supplement: Supplementary file 1 — Figure S1: Monthly delirium consultations to the psychiatric liaison team showed a decreasing trend following sleep medication formulary implementation. [file NPR2-45-e70054-s001.pdf]

Supplementary_Fig1

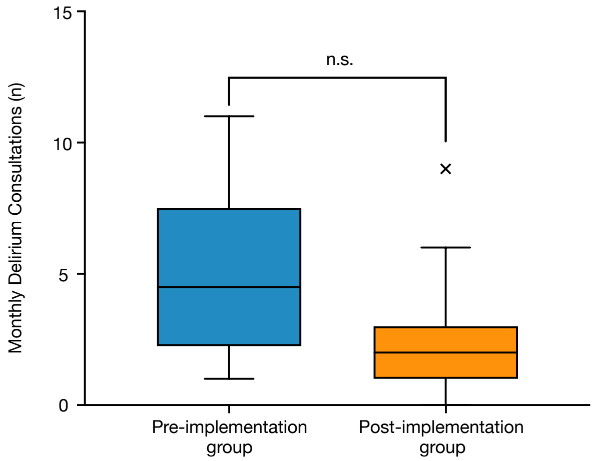

Monthly delirium consultations to the psychiatric liaison team showed a decreasing trend following sleep medication formulary implementation. Pre-implementation group: April 2023 to March 2024 (n = 12 months); Post-implementation group: April 2024 to March 2025 (n = 12 months). Data presented as median with interquartile range (IQR). Whiskers represent 1.5× IQR. n.s., not significant by Mann–Whitney *U*-test.
