# Supplementary material for: Effect of Lemborexant‐Based Sleep Medication Formulary on Benzodiazepine Reduction and Clinical Outcomes: A Single‐Center Retrospective Study
Source: Neuropsychopharmacol Rep. 2025 Sep 8;45(3):e70054. doi: 10.1002/npr2.70054 (PMC12416910; doi:10.1002/npr2.70054)
Supplement: Supplementary file 2 — Table S1: Changes in hypnotic selection in clinical pathways and prescription sets. [file NPR2-45-e70054-s002.docx]

**Supplementary Table 1.** Changes in hypnotic selection in clinical pathways and prescription sets.

| **Hypnotics** | **Pre-implementation group**  n (%) | **Post-implementation group**  n (%) |
| --- | --- | --- |
| Etizolam 0.5 mg | 47 (45.2) | — |
| Zolpidem 5 mg | 26 (25.0) | — |
| Rilmazafone 1 mg | 25 (24.0) | — |
| Nitrazepam 5 mg | 6 (0) | — |
| Lemborexant 5 mg | — | 104 (100.0) |
